# P-663. Acute Respiratory Infection Outbreaks in Long-term Care Facilities in Kyoto, Japan : A 2024/25 Surveillance Report

**DOI:** 10.1093/ofid/ofaf695.876

**Published:** 2026-01-11

**Authors:** Miki Nagao, Yasufumi Matsumara, Yusuke Tsuda, Koh Shinohara, Yasuhiro Tsuchido, Masaki Yamamoto

**Affiliations:** Kyoto University Graduate School of Medicine, Kyoto, Kyoto, Japan; Kyoto University Graduate School of Medicine, Kyoto, Kyoto, Japan; Kyoto University Graduate School of Medicine, Kyoto, Kyoto, Japan; Kyoto University Graduate School of Medicine, Kyoto, Kyoto, Japan; Kyoto University Graduate School of Medicine, Kyoto, Kyoto, Japan; Kyoto University Graduate School of Medicine, Kyoto, Kyoto, Japan

## Abstract

**Background:**

During the COVID-19 pandemic, many long-term care facilities (LTCFs) experienced outbreaks, contributing to healthcare system strain in Japan, similar to trends observed in other countries. However, no systematic surveillance system targeting elderly care facilities has been established in Japan, and the overall burden remains unclear. In this study, we conducted the first acute respiratory infection (ARI) surveillance targeting all LTCFs in Kyoto City to clarify the occurrence and characteristics of outbreaks.

**Methods:**

ARI surveillance was conducted from April 2024 to March 2025 across 132 facilities (with 100% participant rate), covering approximately 8,500 residents. Pathogen identification was performed at Kyoto University using antigen tests or multiplex PCR assays. In this study, we summarized outbreaks involving five or more residents per facility.

**Results:**

During the study period, a total of 89 outbreaks were recorded. The breakdown was as follows: COVID-19 accounted for 79 outbreaks involving 1,263 individuals (0.41 cases per 1,000 bed-days); influenza was identified in 5 outbreaks involving 115 individuals (0.037 cases per 1,000 bed-days); and respiratory syncytial virus (RSV) infection was identified in 3 outbreaks involving 58 individuals (0.019 cases per 1,000 bed-days). Additionally, outbreaks caused by parainfluenza virus, enterovirus, and seasonal coronaviruses were observed. Attack rates ranged from 12% to 42%. The hospitalization rate during COVID-19 outbreaks was 14.1%, compared to 3.4% for influenza and 8.8% for RSV infections. Given that approximately 650,000 residents live in LTCFs nationwide, extrapolation of our surveillance data suggests that outbreaks occur in 7000 LTCFs, and that over 10,000 residents may require hospitalization annually due to outbreaks of acute respiratory infections.

**Conclusion:**

Even five years after the onset of the COVID-19 pandemic, acute respiratory infections, including COVID-19, continue to impose a significant burden on LTCFs. Further research is needed to explore the impact of vaccination status, facility characteristics, and medical care provision within facilities on clinical outcomes.

**Disclosures:**

Miki Nagao, MD, PhD, Beckman Coulter: Research support for a collaborative project|Precision System Science: Research support for a collaborative project Yasufumi Matsumara, MD, PhD, Beckman Coulter: Research support for a collaborative project|Precision System Science: Research support for a collaborative project

